# Comparison between the euglycemic-hyperinsulinemic clamp and the use of proxies for determination of insulin sensitivity in horses

**DOI:** 10.1186/1751-0147-57-S1-O3

**Published:** 2015-09-25

**Authors:** Johan Bröjer, Sanna Lindåse, Cecilia Müller, Katarina Nostell

**Affiliations:** 1 Department of Clinical Sciences, Swedish University of Agricultural Sciences, Uppsala, Sweden; 2 Department of Animal Nutrition and Management, Swedish University of Agricultural Sciences, Uppsala, Sweden

## Background

Accurate quantification of insulin resistance is important for diagnosing and determining the efficacy of treatment in patients with equine metabolic syndrome (EMS). The euglycemic-hyperinsulinemic clamp (EHC) is a gold-standard method for measuring insulin sensitivity (IS) but the complexity of the technique limits its use in research. The use of proxies based on fasting values offers an attractive alternative.

## Objectives

To evaluate the validity of proxies calculated from basal plasma glucose and insulin concentrations that predict insulin sensitivity measured with the EHC in the horse.

## Material and methods

The EHC was conducted in 29 horses of different breeds with a wide range of IS including 12 patients diagnosed with EMS. Basal plasma and insulin concentrations were determined from blood samples collected prior to the EHC. In addition, the ability of proxies to detect changes in IS during a weight gain study in nine Standardbred horses was also included.

## Results

Insulin sensitivity indexes calculated from the EHC (M-value and M/I-value) were correlated (r2 = 0.47 – 0.71) to quantitative insulin sensitivity check index (QUICKI) and to the reciprocal of the square root of insulin (RISQI) as well as to the homeostatic model assessment of insulin resistance (HOMA-IR). The proxies were unable to accurately predict changes in IS during the weight gain study.

## Conclusion

Proxies for screening of IS in the horse may be useful but their ability to detect diet-induced variations in IS appears to be limited.

